# Signaling Crosstalks Drive Generation and Regeneration of the Thymus

**DOI:** 10.3389/fimmu.2022.920306

**Published:** 2022-06-06

**Authors:** Marco Rosichini, Marialuigia Catanoso, Isabella Screpanti, Maria Pia Felli, Franco Locatelli, Enrico Velardi

**Affiliations:** ^1^Department of Pediatric Hematology and Oncology, Cell and Gene Therapy, Bambino Gesù Children’s Hospital, IRCCS, Rome, Italy; ^2^Department of Molecular Medicine, Sapienza University of Rome, Rome, Italy; ^3^Department of Experimental Medicine, Sapienza University of Rome, Rome, Italy; ^4^Department of Maternal and Child Health, Sapienza University of Rome, Rome, Italy

**Keywords:** immune reconstitution, thymus, T cells, immune-senescence, thymic epithelial cells

## Abstract

Optimal recovery of immune competence after periods of hematopoietic insults or stress is crucial to re-establish patient response to vaccines, pathogens and tumor antigens. This is particularly relevant for patients receiving high doses of chemotherapy or radiotherapy, who experience prolonged periods of lymphopenia, which can be associated with an increased risk of infections, malignant relapse, and adverse clinical outcome. While the thymus represents the primary organ responsible for the generation of a diverse pool of T cells, its function is profoundly impaired by a range of acute insults (including those caused by cytoreductive chemo/radiation therapy, infections and graft-versus-host disease) and by the chronic physiological deterioration associated with aging. Impaired thymic function increases the risk of infections and tumor antigen escape due to a restriction in T-cell receptor diversity and suboptimal immune response. Therapeutic approaches that can promote the renewal of the thymus have the potential to restore immune competence in patients. Previous work has documented the importance of the crosstalk between thymocytes and thymic epithelial cells in establishing correct architecture and function of thymic epithelium. This crosstalk is relevant not only during thymus organogenesis, but also to promote the recovery of its function after injuries. In this review, we will analyze the signals involved in the crosstalk between TECs and hematopoietic cells. We will focus in particular on how signals from T-cells can regulate TEC function and discuss the relevance of these pathways in restoring thymic function and T-cell immunity in experimental models, as well as in the clinical setting.

## Introduction

Optimal immune recovery after periods of hematopoietic insults is key to reestablish patient immune competence and sustain response to vaccines, pathogens and tumor antigens. This is particularly relevant for patients receiving high doses of chemotherapy or radiotherapy, for instance, associated with the conditioning regimen employed in preparation to hematopoietic cell transplantation (HCT). These patients experience profound and prolonged periods of lymphopenia, which can be associated with an increased risk of developing life-threatening infections and, in cancer patients, tumor relapse. In fact, infections and relapse have been inversely correlated with the degree of immune reconstitution and account for greater than 50% of mortality after allogenic HCT (allo-HCT) (1–5).

The thymus represents the primary organ responsible for the maturation and differentiation of a broad pool of *naïve* T cells capable of recognizing an extremely large array of pathogens and tumor antigens. The process of T-cell development involves the migration of bone marrow-derived T-cell progenitors through the thymus and requires physical contact between developing thymocytes and the supporting thymic stromal microenvironment which consists of thymic epithelial cells (TECs), macrophages, endothelial cells (ECs), fibroblasts and dendritic cells (DCs) (6–8). Multiple developmental pathways, including Notch, Sonic Hedgehog, and WNT coordinate this complex hierarchical process (9–12). Despite its crucial role in generating T cells, thymic function may be profoundly impaired by acute insults, such as that caused by infections, stress, chemotherapy and radiotherapy. Delayed or defective recovery of thymic function has been associated with adverse clinical outcomes in patients receiving allo-HCT (13–18). Thymic function progressively declines with age, a well-known physiological process known as thymic involution (19). Age-associated thymic involution limits the recovery of thymic function after acute insults and significantly contributes to the decline of T-cell receptor (TCR) diversity in older individuals (20, 21). As a direct consequence, older patients are more prone to bacterial and viral infections and, possibly, to tumor antigen escape. The identification of clinical strategies that can restore thymic function and enhance immune reconstitution represent a major clinical need.

Through the mechanistic understanding of the molecules and pathways driving the maintenance of thymic function and its recovery after insults, several potential regenerative targets have been identified. They include growth factors (such as bone morphogenetic protein 4, stem cell factor, kit ligand and keratinocyte growth factor), the modulation of hormones (such as the inhibition of sex steroids and the use of growth hormone, insulin-like growth factor-1 and ghrelin), cytokines (such as interleukin (IL)-7, IL-12 and IL-21), chemokines (such as CXCL12/CXCR4) and the adoptive transfer of preformed T-cell progenitors, as well as *ex vivo* expanded thymus-derived endothelial cells (22). However, at present, none of these approaches is approved as a standard therapy to enhance thymic function and immune reconstitution.

## Thymic Crosstalk Regulates Tissue Maintenance and Its Regeneration

Thymic crosstalk, a set of reciprocal regulations between thymocytes and the thymic environment, is critical to orchestrate thymocyte and TEC development, as well as to start thymic recovery after periods of stress or immunological injuries (23). Thymic epithelium represents a predominant stromal cell population within the thymus, which is classically divided into two subsets based on their spatial distribution and specialized function: cortical TECs (cTECs) and medullary TECs (mTECs) are responsible for positive and negative selection of thymocytes, respectively (10).

cTECs are critical for fate commitment, expansion, and positive selection of the developing thymocytes. On the other hand, mTECs are involved in the negative selection and maturation of thymocytes (2–4). mTECs can be further divided based on the expression of MHCII and additional molecules, such as CD40 and CD80/86. Within thymic microenvironment, while innate lymphoid cells (ILCs), endothelial cells and fibroblasts are mostly resistant to damage (24–26), thymic epithelium is particularly sensitive to the effects of chemotherapy and radiotherapy, with the MHCII^high^ mTEC subset representing the population most sensitive to insults, likely due to the high proliferative rate of these cells (27, 28).

TECs play a fundamental role in the development and selection of T cells providing key thymopoietic signals, including Interleukin-7 (IL-7), Notch-ligand Delta Like 1 and 4, as well as self-peptide–MHC complex. On the other hand, the maturation and maintenance of TECs is closely dependent on instructive signals provided by the bone marrow-derived lymphoid component. Indeed, thymocyte-derived signals are indispensable for the appropriate development and spatial organization of cTEC and mTEC subsets during late fetal development and adult life as revealed through the use of different genetic mouse models (29–31). *Tcra* KO and Zeta-chain-associated protein kinase 70 (*Zap70*) KO mice, in which thymocyte development is blocked at the double positive (DP) stage, showed severely impaired thymic medulla organization (32, 33). Similarly, *Recombination activating gene (Rag)*1 KO and *Rag2* KO mice, in which thymocyte development is arrested at the double negative (DN) 3 stage, showed impaired medulla formation. Transgenic mice expressing high copies of the human CD3 epsilon molecule, which display a block at the DN1 stage of differentiation, showed impaired cortical thymic function and disrupted thymic architecture (34, 35). Importantly, transplantation of T-cell depleted bone marrow cells in severe combined immunodeficiency (SCID) mice, restored thymic architecture organization (36). In addition, the transfer of mature T cells into SCID mice promoted the recovery of the medullary epithelial structure, providing evidence that the regenerative signals on thymic epithelium can be instructed by both progenitor and mature T cells (37).

Interestingly, data suggesting that the infusion of mature T cells can boost thymic and immune recovery come also from clinical studies in which patients received allo-HCT followed by the adoptive transfer of donor T cells. Vago et al. demonstrated that the transfer of donor T cells genetically engineered to express the Herpes Simplex Virus thymidine kinase suicide gene (a safety switch system to be activated in case of graft-versus-host disease, GvHD) induced improved thymic function, as demonstrated by increased levels of T-cell receptor excision circles (TRECs) and recent thymic emigrants (RTEs) (38). Using chest tomography scans, this study also demonstrated that patients infused with modified T cells showed enlargement of active thymic tissue when compared to pre-transplant levels (38). In addition, recent observations collected at our center suggested that patients receiving donor T-cells genetically modified with the inducible Caspase 9 suicide gene showed rapid recovery of thymic function evaluated by the quantification of TRECs in patient peripheral blood (39). Data on enhanced immune recovery after the infusion of mature T-cells in patients, come also from studies in which the adoptive transfer of virus-specific T-cells generated a broad enhancement of the T-cell immunity (40, 41). The beneficial effect of the infused mature T cells on thymic function is likely to be transient in nature, but sufficient to provide regenerative signals, which result in faster recovery of thymic structure and accelerated immune reconstitution post damage. Nevertheless, it remains to be explored the long term persistence of these effects. The characterization of the underlying mechanisms of such effects could be of valuable importance to reveal pathways crucial for the regeneration of the human thymus that can be exploited to develop immune boosting therapies.

## Regenerative Pathways

**Figure 1:**
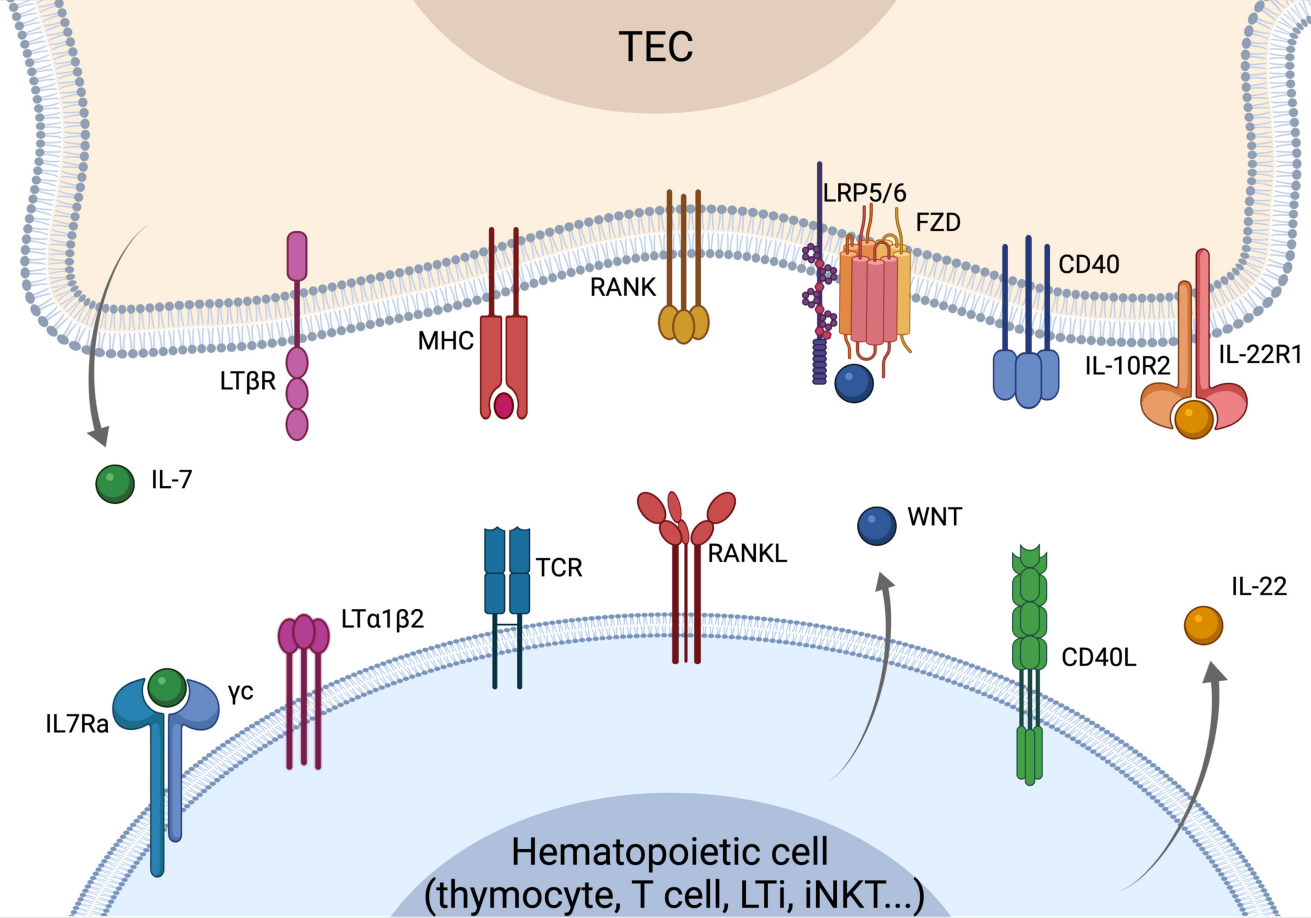
Overview of the crosstalk signals driving TEC development, differentiation and regeneration. Recover of thymus function is strictly dependent on the crosstalk signals between TECs and cells of the hematopoietic compartment. LTα and RANKL are mostly provided by SP thymocytes leading to mTEC maturation and differentiation. RANKL is overexpressed by CD4 thymocytes and LTi cells upon insults and can drive the recovery of thymus function. WNT signaling regulates TEC proliferation and homeostasis, while CD40L is involved in TEC maturation and proliferation. LTi- and T-cells-derived IL-22 is key in sustaining TEC proliferation and thymus recovery upon insults. IL-7 is mostly produced by cTEC acting as a key mediator of thymocyte maturation and proliferation. Elements of the figure were generated using Biorender.com.

In this review, we will analyze the signals involved in the crosstalk between TECs and T-cells, looking beyond the process of thymocyte maturation and exploring how signals from T cells can regulate TEC function (**Figure 1**). Although several pathways (including Notch and Hedgehog) are known to have pivotal roles in T-cell and TEC development, we will highlight crosstalk signals described to regulate thymic function and T-cell immunity postnatally in experimental models, as well as in the clinical setting.

## RANKL

Receptor activator of nuclear factor kappa B ligand (RANKL) is a TNF superfamily member encoded by *Tnfsf11* gene in mouse (42). Although a soluble form of RANKL (sRANKL) exists, this factor is expressed as a type II transmembrane protein whose ectodomain specifically interacts with its cognate receptor RANK (encoded by Tnfrsf11a). Thus, RANK-RANKL signaling is mostly mediated by the physical interaction of different cell types. RANK stimulation results in both canonical and non-canonical NF-kB signaling, together with MAPK activation (43). These events lead to the upregulation of genes involved in proliferation, survival and differentiation, thus resulting in pleiotropic effects on human physiology. First identified as a key component of bone metabolism, RANKL was then characterized as a crucial mediator in both organ development and immunity (44). In fact, despite having normal splenic architecture, *Tnfrsf11a* KO mice show null lymph-nodes organogenesis, while *Tnfsf11* KO mice show reduced thymic size and block of thymocyte maturation between DN3 and DN4 stage of differentiation (45, 46).

Within the thymus, RANK is expressed by subsets of mTECs residing in both Aire^+^ and Aire^-^ subpopulations (47, 48). On the other hand, RANKL is mostly provided by CD4 SP thymocytes and LTi cells, while CD8 SP thymocytes and invariant natural killer T (iNKT) cells contribute for the presentation of RANKL to a lesser extent (48–50). In this context, cell-cell interactions are of paramount importance in controlling central tolerance and T-cell production, as RANK signaling stimulates Aire^+^ mTEC maturation in concert with CD40 and LTα pathways (47, 51). Importantly, Aire^+^ mTEC^HI^ cells are also the primary cell population responsible for the production of osteoprotegerin (OPG) in the thymus, a soluble decoy receptor for RANKL encoded by *Tnfrsf11b* (52). OPG binding to RANKL inhibits its interaction with RANK. In fact, thymus tissues from *Tnfrsf11b* KO mice show increased mTEC cellularity (50, 53).

Besides its roles in thymic physiology, RANK-RANKL pathway is also implied in thymic regeneration upon immunological insults. In fact, RANKL is upregulated in CD4 thymocytes and LTi cells during thymus recovery in mice exposed to sublethal total body irradiation (SL-TBI) (54). On the other hand, a recent report demonstrated that increased CD4 T-cell-mediated RANK signaling in the thymus causes enhanced generation of mTEC. This results in an imbalance of cTEC and mTEC proportion, eventually leading to defective thymopoiesis (55). For its pivotal role in health and disease, the administration of RANKL or RANKL partial agonists has been exploited in mouse models reproducing particular clinical conditions, such as psoriasis and ischemic stroke (54, 56–58). Furthermore, in mouse models of HCT, sRANKL exogenous administration drives TEC regeneration, as demonstrated by increase in cellularity of thymic epithelial progenitor cells, cTEC and mTEC subsets (54). sRANKL-treated mice also showed early homing of lymphoid progenitors in the thymus and T-cell reconstitution (54). Moreover, Desanti et al. showed that stimulation of mTEC progenitors with RANK agonistic antibodies resulted in CD40 upregulation, thus suggesting a role in mTEC maturation (59).

## CD40L

CD40L is a transmembrane protein and a tumor necrosis factor (TNF) superfamily component playing key roles in both innate and adaptive immunity (60). CD40L is expressed by activated T and B cells, basophils, monocytes, NK and mast cells and signals through physical interaction with its cognate receptor CD40 (61). The latter is a transmembrane costimulatory receptor firstly identified on B cells as a factor responsible for their activation and proliferation (62). In subsequent studies, CD40 was also reported to be expressed by activated T cells, DCs, fibroblasts, epithelial and endothelial cells (60, 63–66). CD40 signaling drives upregulation of co-stimulatory molecules, cytokine production and cross-presentation of the antigen in DCs, thus promoting DC-mediated T-cell activation (60, 67). Moreover, it was shown that CD8 and CD4 T cells directly communicate through CD40-CD40L interaction and this pathway is indispensable for the generation of CD8 T-cell memory (66).

As CD40-CD40L axis plays a crucial function in antigen presenting cell (APC) regulation, several studies investigated the role of CD40 signaling within the thymus in the context of T-cell development and selection, and self-tolerance induction (68). Here, similarly and in synergy with RANKL, CD40L stimulates mTEC maturation in the postnatal thymus, with both *Cd40* KO and *Cd40lg* KO mice showing a reduction in mTECs without affecting cTEC compartment (27, 51). On the other hand, Dunn et al. produced transgenic mice expressing CD40L cDNA under the control of the proximal *lck* promoter (69). These mice carrying *Cd40lg* overexpression in thymocytes showed alterations in organ architecture, with an abnormal mTEC proportion and reduction in thymus cortex (69).

Within the thymic medulla, RANKL and CD40L are upregulated in CD4 single positive (SP) thymocytes, this finding suggesting a key role of CD4 SP in regulating mTEC maturation and homeostasis (50, 59, 70). However, flow-cytometry analyses highlighted a great heterogeneity within CD4 SP population, with CD25^-^CD4^+^TCRβ^high^ thymocytes showing the highest RANKL positivity during the early SP stage (CD69^+^), while being mostly CD40L^+^ in subsequent maturation steps (CD69^-^) (59). This temporal regulation of TNF family ligands expression in thymocytes is paralleled by a RANKL-dependent CD40 upregulation in mTECs, eventually leading to mTEC proliferation and maturation (59).

## Lymphotoxin-α

Lymphotoxin-α (LTα) is another member of TNF superfamily that was originally identified as a soluble factor secreted by lymphocytes having cytotoxic effects on tumor cells (71, 72). Subsequent studies showed that, besides its soluble homotrimer (LTα3) form, LTα could associate with the transmembrane protein LTβ resulting in the membrane-bound heterotrimer LTα1β2 (73). The latter signals through cell-cell interactions with its cognate receptor LTβR, resulting in both canonical and non-canonical activation of NF-kB pathway (74, 75). This signaling has several implications in immunity including the regulation of lymphoid organ development. In fact, both *Lta*, *Ltb* and *Ltbr* KO mice show similar phenotypes lacking lymph nodes and Peyer’s patches, and abnormal splenic architecture (76–78).

Besides activated T and B cells, LTα1β2 is also expressed by NKs and type 3 ILCs (ILC3). On the other hand, LTβR is mainly expressed by epithelial and endothelial cells among macrophages and DCs (72).

Within the thymus, LTβR is expressed by the entire stromal compartment, especially by TECs, while LTα1β2 is mostly provided by single positive thymocytes (70, 79). Here, LTβ or LTβR deficiency leads to aberrant mTEC development and altered medulla organization (79–82). In particular, it was shown that LTα/LTβR signaling mediated by mature thymocytes is indispensable for the generation of terminally differentiated mTECs, as demonstrated by involucrin expression (83).

Besides its role in steady state, LTα is also important during insult recovery, as demonstrated by the fact that both *Lta* and *Ltbr* KO mice show impaired thymic recovery in *in vivo* models of HCT (47, 84). Upon SL-TBI, LTα1β2 upregulation is induced in radio-resistant LTi cells leading to thymic recovery through the stimulation of TEC proliferation and survival (54). On the other hand, LTα/LtβR signaling is also implied in T-cell progenitors homing and mature T-cells egress from the thymus in both steady state and HCT settings (84–86). For these reasons, LtβR agonistic antibody administration following HCT has been evaluated in mouse models, leading to an increase in thymic output and immune reconstitution (85).

## Interleukin-7

IL-7 is a stromal-derived, non-redundant cytokine having a key role in regulating immunity and immune reconstitution (87). The active form of human IL-7 is a glycoprotein of 25 kDa that is mainly produced within the lymphoid organs and that signals through the IL−7 receptor (IL−7R) (2). The latter is a hetero−dimer consisting of IL−7Rα and the common cytokine receptor γ−chain (γc). Triggering of the receptor mediates anti−apoptotic and co−stimulatory proliferative signals, mostly on T- and B-cell lineages (88). In the thymus, IL-7 is primarily produced by TECs and fibroblasts (22). Using a IL-7 reporter mouse, it has been shown that TECs expressing high levels of IL-7 reside within a subset of cTECs defined as CD205^+^Ly51^+^CD40^low^ (89). Cooperatively with Notch1, IL-7 provides proliferative signals to DN and DP thymocytes (90) and also sustains the recombination of the T-cell receptor γ−chain (TCRγ) locus (87). On the other hand, besides receiving maturation signals throughout their development, thymocytes control mTEC gene expression and differentiation, thus regulating the formation of a proper thymic microenvironment architecture (50, 70). For instance, thymocytes can downregulate *Il7* expression by TECs in a negative feedback fashion (91). In fact, lymphopenic *Rag2 Il2rg* double KO mouse strain shows a markedly increased proportion of IL−7^+^ TECs compared to WT mice (91). IL-7R-deficient mice show defective thymic microenvironment, especially in corticomedullary structure, and reduced mTEC development (92, 93). While this phenotype is most likely due to a failure of the crosstalk normally provided by IL-7-dependent thymocytes and other cells of the hematopoietic lineage, a possible direct impact of IL-7 on thymic stromal cells is currently unknown. Interestingly, as discussed above, Vago et al. observed that serum levels of IL-7 peaked after every infusion of donor T-cells in transplanted patients, this suggesting that mature T-cells may induce IL-7 production, although the underlying mechanism is still largely obscure (38).

In the periphery, IL-7 has a key role in T-cell homeostatic proliferation and its production is tightly regulated, as the levels of IL-7 in the peripheral blood increase during lymphopenia remaining high until T-cell pool returns to steady state conditions (18, 94, 95). Given its crucial role in T-cell homeostasis, exogenous administration of IL-7 has been tested in several clinical conditions (87). In the context of HCT, IL-7 administration drives both CD4 and CD8 T-cell expansion, and this phenomenon is accompanied by an increase of TCR repertoire diversity (96). Most recently, IL-7 administration has been used in a murine model of age-related lymphopenia. Aged mice were subjected to IL-7 treatment and both numbers of CD4 and CD8 *naïve* T-cells in spleen and lymph nodes rose to levels similar to those observed in adult mice (97).

## Interleukin-22

IL-22 is a monomeric cytokine released as a 179 amino acid monomeric protein (98). As IL-7, IL-22 is a non-conventional cytokine targeting stromal rather than hematopoietic compartment. In fact, the main targets of IL-22 are epithelial cells and fibroblasts within the thymus, liver, kidneys, lung and pancreas (99). On the other hand, the main contributors for IL-22 production are αβ and γδ T-cells, as well as ILCs, although fibroblasts, neutrophils and macrophages are also reported as secondary sources of IL-22 (99–104).

While systemic expression of IL22 is low during steady state, its production is induced upon negative stimuli, such as tissue injury and inflammation (99). During these pathologic conditions, IL-22 exerts controversial effects, being involved in both epithelial tissue regeneration and upregulation of different inflammatory mediators, including TNF, IL-6 and LPS-binding protein (105–107).

Within the thymus, IL-22 is involved in stromal regeneration following insults. In fact, IL-22 upregulation occurs in thymus-resident lymphoid tissue inducer (LTi) cells in mice exposed to SL-TBI (107). In turn, IL-22 production acts directly on mTEC compartment, providing proliferation and survival signals to the damaged tissue (107). Besides endogenous IL-22 production in injured thymus, recent findings demonstrated that exogenous administration of IL-22 could also promote faster thymic recovery. In fact, murine models of HCT showed that donor-derived T-cells are a major contributor for IL-22 production upon transplantation, leading to TEC proliferation and thymus recovery (108, 109). Moreover, exogenous IL-22 administration accelerates thymic regeneration after insults (107, 109).

Although IL-22 administration is currently being evaluated for the treatment of several conditions, only few trials are exploring the infusion of IL-22 or their agonists in the HCT setting (NCT02406651, NCT04539470). While these studies are primarily focused on acute GvHD treatment or prevention, the recent results herein reviewed suggest the possibility to use IL-22 in restoring thymic function during the first period after the transplant.

## WNT

WNT-signaling plays an important role during thymic development and in the maintenance of its function in adult life (11). In humans, 19 different WNT family members have been identified along with 15 WNT receptors and coreceptors. WNT regulates the stabilization of β-catenin which, in the absence of any WNT signaling, is degraded in a cytoplasmatic “destruction complex” consisting of glycogen synthase kinase 3β (GSK), adenomatous polyposis coli (APC), axis inhibition protein (AXIN) and casein kinase (CK). After the binding of WNT to a member of the Frizzled receptor family and its coreceptors low-density lipoprotein-receptor related proteins (LRP) 5 and 6, the β-catenin is no longer degraded leading to its accumulation, activation and translocation to the cell nucleus where it regulates downstream transcription factors of the TCF/LEF family. The crucial role of WNT in the thymus has been demonstrated in several genetic models. *Tcf-1* KO mice showed altered T-cell differentiation with a partial block at the double negative and immature single positive stages (110). Mice carrying a constituency active form of β-catenin in TECs show altered thymic organogenesis, reduced TEC proliferation and loss of TEC identity (111). The inhibition of WNT signaling through the forced expression of the canonical WNT inhibitor DKK1 leads to loss of TEC progenitors and thymic degeneration (112). Downregulation of WNT signaling has been also linked to the age-associated involution of the human thymus (113).

While stromal cells, such as TECs, are the major producers of WNT family members, cells of the hematopoietic lineage can also express WNTs. WNT proteins, such as WNT4 and WNT5b, expressed by TECs and thymocytes sustain the proliferation of TECs, which is partially achieved by increasing the expression of the key thymopoietic factor FoxN1 (114–116). Upregulation of FoxN1 expression represents a major step towards the regeneration of thymic function. Previous studies demonstrated that FoxN1 and its downstream genes are upregulated during the endogenous process of thymic reconstitution after sublethal dose of radiation (24). Importantly, induction of FoxN1 expression alone is sufficient to reverse thymic involution and regenerate the organ in mice (117). Together, these data demonstrate that the levels of FoxN1 tightly control thymic regeneration and the identification of factors regulating its expression could have a strong rationale for thymic boosting approaches. Whether mature T-cells can express members of the WNT family and induce the upregulation of FoxN1 in TECs when transferred *in vivo* would represent an interesting regenerative approach to investigate.

## Conclusions

Several strategies have been proposed to restore thymic function after injuries and insults. Among these, the administration of chemokines and growth factors have been explored in several preclinical mouse studies displaying very promising results. However, when transferred to the clinic, the same strategies have shown modest regenerative potential. Until now, increasing thymic function and T-cell production remains a major challenge for the treatment of several conditions, especially in the early phase following HCT. Besides the HCT setting, boosting thymic function is of paramount importance for the treatment of other T-cell deficiencies associated with pathological, as well as physiological conditions. Thymic involution is a well-known phenomenon associated with a progressive decline of thymic size and output with age which paralleled with a decrease in immune surveillance in the elderly (118–120). Therapeutic approaches that can promote thymic function in older individuals can increase peripheral T-cell diversity, enhance the immunity against pathogens and response to vaccines, and, possibly, reduce the risk of malignancy through better immune-surveillance mechanisms against transformed cells. As previously discussed, the work by Vago et al. demonstrated that the infusion of mature donor T cells can rejuvenate the thymus of adult transplanted patients (aged 17-66). Whether a similar approach can restore TEC functionality in older individuals, in which the residual thymic tissue is limited (121) remains an avenue to be explored. On the other hand, it is highly unlikely that the same approach can mediate beneficial effects in restoring thymic function and the process of T-cell development in patients with intrinsic genetic defects which alter TEC function, for instance as a consequence of FoxN1 deficiency in patients affected by the nude/severe combined immunodeficiency. In fact, these defects cannot be mitigated by changes in the hematopoietic compartment as suggested by the inefficacy of bone marrow transplantation in these patients (122).

## Author Contributions

MR, MC, IS, MF, FL and EV wrote, drafted, and edited the manuscript. All authors contributed to the article and approved the submitted version.

## Funding

EV was supported by grants from the Amy Strelzer Manasevit Research Program; the Italian Association for Cancer Research (AIRC); and the Italian Ministry of Health (“Ricerca Corrente”). FL was supported by grants from AIRC (Special Program Metastatic disease: the key unmet need in oncology 5 per mille 2018 Project Code 21147 and Accelerator Award 2017 INCAR); Ministero dell’Istruzione, dell’Università e della Ricerca, PRIN ID 2017 WC8499_004; Ministero della Salute, RF-2016-02364388. IS and MPF, THYMINNOVA (IP-2020-02-2431).

## Conflict of Interest

The authors declare that the research was conducted in the absence of any commercial or financial relationships that could be construed as a potential conflict of interest.

## Publisher’s Note

All claims expressed in this article are solely those of the authors and do not necessarily represent those of their affiliated organizations, or those of the publisher, the editors and the reviewers. Any product that may be evaluated in this article, or claim that may be made by its manufacturer, is not guaranteed or endorsed by the publisher.
